# The study on potential pharmacological mechanism of semen strychny against glioma via network pharmacology analysis, molecular docking, and experimental verification

**DOI:** 10.1097/MD.0000000000046392

**Published:** 2025-12-19

**Authors:** Wenqu Jiang, Liang Li, Qiwei Huang, Guofeng Zhu, Zhixiong Zhang, Yi Zhang, Xiang Gao

**Affiliations:** a Department of Neurosurgery, Jiujiang University Affiliated Hospital, Jiujiang, Jiangxi, China; b Department of Neurosurgery, Lushan People’s Hospital of Jiangxi Province, Jiujiang, Jiangxi, China.

**Keywords:** experimental verification, glioma, molecular docking, network pharmacology, semen strychni

## Abstract

**Background:**

Semen strychni is commonly used to treat gliomas in China. However, the underlying mechanism of action of semen strychni in glioma treatment remains unknown. Using network pharmacology and molecular docking, we investigated the probable pharmacological mechanisms of semen strychni in glioma treatment.

**Methods:**

The active components of semen strychni were retrieved from the Traditional Chinese Medicine Systems Pharmacology database. Using the Cytoscape program created the active component-target network diagram of semen strychni. The GeneCards, DisGeNET, and Online Mendelian Inheritance in Man databases were used to compile the list of targets related to glioma. Using the STRING database created a protein–protein interaction network of popular drugs and disease targets. For Gene Ontology enrichment and Kyoto Encyclopedia of Genes and Genomes pathway analysis of common targets, the Database for Annotation, Visualization and Integrated Discovery database was utilized. In order to identify the main targets of semen strychni in the treatment of glioma, a target-pathway interaction network was built. The molecular docking of critical and crucial components was done using AutoDock. Glioma samples from the Cancer Genome Atlas and Human Protein Atls databases were extracted to detect the mRNA and protein expression levels of ADRB1 and ADRB2 and analyze the correlation between expression and survival of glioma patients. 3-(4,5-dimethylthiazol-2-yl)-5-(3-carboxymethoxyphenyl)-2-(4-sulfophenyl)-2H-tetrazolium, Transwell, and wound healing assays validate the effect of stigmasterol on the malignant progression of glioma cells.

**Results:**

The core components of semen strychni used in the treatment of glioma included stigmasterol, (S)-stylopine, isobrucine, icaride A, and isostrychnine N-oxide (I), which may operate on core targets such as ADRB1, ADRB2, CHRM3, ADRA1A, and ESR1. The significant pathways discovered using Kyoto Encyclopedia of Genes and Genomes enrichment analysis were chemical carcinogenesis-receptor activation, calcium signaling pathway, and pathways in cancer. The outcomes of the molecular docking demonstrated a strong affinity between the central targets and elements. Data from the Cancer Genome Atlas and Human Protein Atls databases indicate that ADRB1 and ADRB2 are expressed in glioma, and high expression of ADRB2 may predict a good outcome. In vitro experimental results indicate that stigmasterol can inhibit the malignant progression of U251 cells.

**Conclusion:**

This article discusses the probable mechanism of action of semen strychni in the treatment of glioma, which can serve as a theoretical basis and evidence for subsequent experimental investigations.

## 1. Introduction

More than 80% of all primary malignant tumors affecting the central nervous system are gliomas, the most prevalent and deadly malignant tumor of the nervous system.^[1]^ The age-adjusted incidence rates for all gliomas range from 4.67 to 5.73 per 100,000 people, showing an increase in glioma incidence. Glioblastoma is the most prevalent and deadly form of adult glioma, and its age-adjusted incidence varies from 0.59 to 3.69 per 100,000 people.^[2]^ With an average annual age-adjusted incidence rate of 6.0 per 100,000 people between 2010 and 2014, glioma is the most prevalent kind of malignant brain tumor in the United States.^[3]^ The cause of glioma is unknown, and both environmental and genetic factors may increase the risk of developing this disease.^[4,5]^ Current treatments include surgical resection, chemotherapy, and radiation. However, their effectiveness is limited.^[6]^ Therefore, it is essential to understand the molecular etiology of glioma growth and develop new treatment techniques.

Semen strychni is a traditional Chinese medicine commonly used in clinical practice. It is a bitter cold that can relieve pain and swelling through the liver and spleen channels.^[7]^ Recent studies have shown that strychnine effectively antagonized tumor development. Many reports have shown that strychnine in semen strychni enhances cellular immune function, inhibits tumor angiogenesis, reduces tumor cell proliferation, and promotes cell apoptosis.^[8,9]^ However, the basic mechanisms underlying the detection and treatment of gliomas remain unclear.

**Figure 1.**
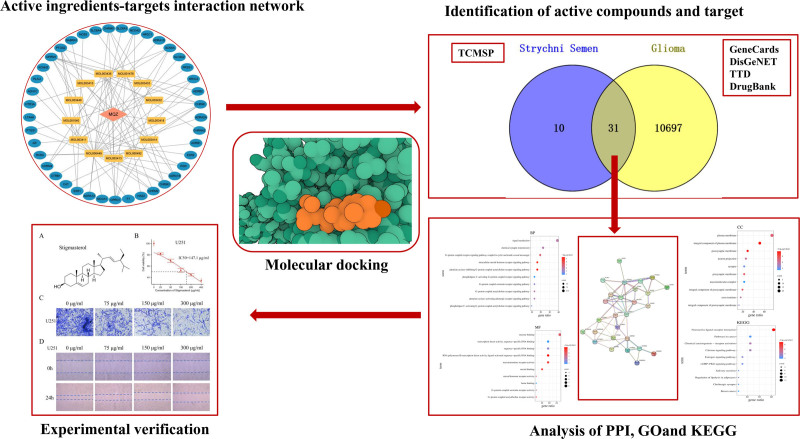
Detailed flow chart of this study.

Network pharmacology is a new, multidisciplinary area that combines computer science and bioinformatics and is based on the systems biology idea. The “multi-component, separate-target, multi-pathway” synergistic interaction between medications, illnesses, and targets may be examined using network pharmacology. It has been crucial for investigating and developing new forms of traditional Chinese medicine as well as understanding the pharmacological and toxicological processes of traditional Chinese medicine.^[10]^ Utilizing the 3 complementary ideas of geometry, energy, and chemical environment, molecular docking employs receptors and ligands with known structures to detect interactions between molecules and predict the best binding mode between molecules. The study of the pharmacological mechanisms of Chinese herbal compound prescriptions, as well as the research into potential targets and action mechanisms of active components in traditional Chinese medicine, all benefit from its great value and potential advantages.^[11]^ This study used network pharmacology, molecular docking techniques, and experimental verification from traditional Chinese medicine to evaluate the potential mechanism of action of semen strychni in the treatment of glioma, providing a theoretical framework for further investigation (Fig. 1).

## 2. Materials and methods

### 2.1. Data source

Traditional Chinese Medicine Systems Pharmacology Database (TCMSP, https://www.91tcmsp.com/),^[12]^ UniProt database (http://www.uniprot.org),^[13]^ GeneCards database (https://www.genecards.org), DisGeNET database (https://www.disgenet.org),^[14]^ Online Mendelian Inheritance in Man (OMIM, https://omim.org/),^[15]^ String database (Version 11.0, https://string-db.org),^[16]^ the Database for Annotation, Visualization and Integrated Discovery (DAVID, https://davidbioinformatics.nih.gov/),^[17]^ Protein Data Bank (PDB, https://www.rcsb.org/). Online drawing tools: Venny (Version 2.1.0, https://bioinfogp.cnb.csic.es/tools/venny), and Bioinformatics (http://www.bioinformatics.com.cn). Software: Cytoscape (version 3.8), AutoDock (V4.2.6), AutoDock Tools (V1.5.6), and PyMOL (V2.4.0).

### 2.2. Identification of active ingredients and targets of strychnine

In order to achieve the criteria of oral bioavailability (OB) > 30% and drug-like > 0.18, the chemical composition of semen strychni was obtained from the TCMSP, and the active components were tested according to ADME (adsorption, distribution, metabolism, and excretion). Following the acquisition of the active components, the TCMSP database was searched for matching targets, and the UniProt database was used to standardize the target name. The active component-target network diagram of semen strychni was created using the Cytoscape program. The essential elements of semen strychni were identified based on the degree value by studying the network function.

### 2.3. Identification of glioma-associated targets

“glioma” (Mesh) was used as the primary search word to obtain targets associated with glioma from the GeneCards, DisGeNET, and OMIM databases. Glioma-associated targets were discovered by integrating the targets collected from the aforementioned database and eliminating duplicates.

### 2.4. Construction of protein–protein interaction network of common target of strychnos and glioma

Using Venny, it was possible to identify the interaction targets between semen strychni and gliomas, and a Venn diagram was created. The STRING database was used to construct a protein–protein interaction (PPI) network between the interaction targets. The organism was “*Homo sapiens*,” and the mode was “Multiple Protein.” The unconnected nodes in the network were concealed, and the minimum necessary interaction score was set to “Highest confidence (0.400).” The other variables stayed the same.

### 2.5. Analysis of Gene Ontology and Kyoto Encyclopedia of Genes and Genomes enrichment

Using the DAVID database, Gene Ontology (GO) enrichment and Kyoto Encyclopedia of Genes and Genomes (KEGG) pathway analyses were carried out on the interaction targets of semen strychni and glioma. The species and background were both set to “*H. sapiens*,” and the Select Identifier was set to “OFFICIAL GENE SYMBOL.” Analyses of the KEGG pathway, biological process (BP), cellular component (CC), and molecular function were also carried out. *P*-values were used to export and order the data. The 10 elements with the lowest *P*-values for each GO enrichment and KEGG pathway were chosen to create an advanced bubble diagram utilizing bioinformatics.

### 2.6. Construction of a common target-pathway interoperability network

In order to create the targets-pathways interaction network, the 10 KEGG pathway components with the lowest *P*-values were imported into the Cytoscape program together with the interaction targets between the drugs and disease targets included in the aforementioned KEGG pathways. The primary semen strychni targets for the therapy of gliomas were determined by examining network function in accordance with degree value.

### 2.7. Molecular docking

The chosen cores and core targets were subjected to a molecular docking procedure called AutoDock. The TCMSP database’s mol2 formats for the core components were downloaded, converted to pdb formats with PyMOL, and then saved in pdbqt forms with AutoDock Tools. From the PDB database, the core targets’ PDB files were obtained. The protein complex with a ligand and a resolution of 3A was chosen. PyMOL was used to carry out water removal, hydrogenation, and original ligand removal. When the atomic type was imported into AutoDock Tools to be saved in pdbqt format, it was configured to be the Assign AD4 type. The active pocket in the protein complex was identified as the location of the initial ligand, and the Lamarckian genetic strategy was used for molecular docking using AutoDock. The optimum docking outcomes were determined using binding free energy. Finally, the PyMOL program was used to illustrate the outcomes.

### 2.8. Expression and prognostic validation of molecular docking targets in the Cancer Genome Atlas and Human Protein Atls databases

Molecular docking targets were validated in glioma samples from the Cancer Genome Atlas database (TCGA, https://portal.gdc.cancer.gov/) for mRNA expression and survival analysis. The Human Protein Atls database (HPA, https://www.proteinatlas.org/) was utilized to search for the expression and distribution of target proteins in normal tissues and gliomas.

### 2.9. Cell culture and drug sources

Glioma cells U251 were purchased from Fuheng Biotechnology (Fuheng, Shanghai, China) and cultured in Dulbecco Modified Eagle Medium supplemented with 10% fetal bovine serum (FBS) and 2-mM L-glutamine. Additionally, streptomycin (100 ng/mL) and penicillin (100 U/mL) were added to the medium, and the cells were incubated at 37℃ in a humidified incubator with 5% CO_2_. Stigmasterol (China, CAS No.: 83-48-7) was acquired from MedChemExpress (Shanghai, China) and dissolved in dimethyl sulfoxide. Before use, these compounds were diluted to specific concentrations in complete medium, ensuring that the final concentration of dimethyl sulfoxide in the given drug was <0.05%.

### 2.10. 3-(4,5-dimethylthiazol-2-yl)-5-(3-carboxymethoxyphenyl)-2-(4-sulfophenyl)-2H-tetrazolium (MTS) assay

Cell viability was evaluated using the MTS method (Promega Kit, Madison), following the manufacturer’s protocol. Briefly, 8000 cells (U251) were seeded in each well of a 96-well plate with 100 μL of medium and treated with various concentrations of drugs. After the specified incubation time, 10 μL of MTS solution (containing 90 μL of serum-free Dulbecco Modified Eagle Medium) was added to each well and incubated at room temperature for 30 minutes. Absorbance was then measured at 490 nm using a Bio-Rad microplate reader from the United States.

### 2.11. Transwell invasion assay

The invasion assay was conducted using a transwell chamber (Millipore, Billerica) containing Matrigel (BD, San Jose). Cells treated with sterols were seeded in the upper chamber with 100 µL of serum-free medium (2 × 10^4^ cells), while the lower chamber contained medium with 20% FBS. After 48 hours of incubation, cells were fixed and stained with crystal violet. Under an optical microscope, the number of invaded cells in 4 randomly selected fields was counted at a magnification of ×100.

### 2.12. Wound healing assay

U251 cells were seeded into a 6-well plate at a density of 3 × 10^5^ cells per well, allowing them to adhere uniformly. When the cell growth reached over 90%, 5 scratches were made at the bottom of the 6-well plate using a 200-μL pipette tip, followed by washing the cells twice with PBS. After removing the scratched cells, the control group was supplemented with 2% FBS medium, while the treatment group received 2% FBS medium at different concentrations. The cells were then incubated at 37°C in a constant temperature incubator for 24 hours, imaged under a microscope, and photographed. The scratch area was calculated using Image J software. This experiment was performed in triplicate.

### 2.13. Statistical analysis

All data parts of the network pharmacology analysis were automated by software or databases, such as the *P*-values for GO and KEGG, which were statistically analyzed automatically by the DAVID database. In addition, experimental data were analyzed using GraphPad Prism 8.0 software (GraphPad, La Jolla) and are presented as mean ± standard deviation.

## 3. Results

### 3.1. The acquisition of effective components and targets of strychnine

**Figure 2.**
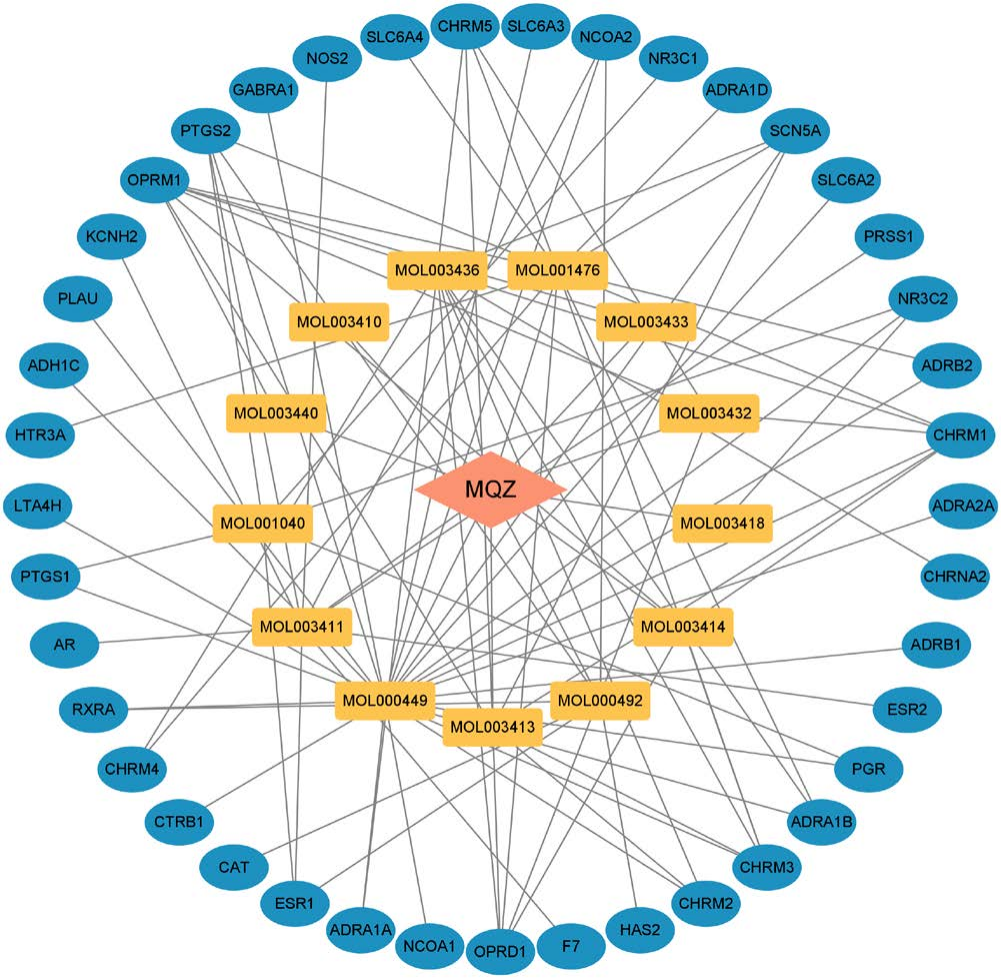
The network of interactions between semen strychni’s active components and targets.

The TCMSP database was utilized to acquire the components and targets of semen strychni, and the inclusion criteria were OB > 30% and drug-like > 0.18, and 13 active components were found after removing duplicates and nontarget components. According to their OB values, the 13 active components were grouped from biggest to smallest (Table 1). Retrieving the information from the TCMSP database resulted in the identification of 41 targets. The names of the targets were standardized using the UniProt database. The active component-target interaction network of semen strychni was shown using Cytoscape software (Fig. 2). According to the degree value of the software’s assessed network function, stigmasterol, (S)-stylopine, isobrucine, icaride A, and isostrychnine N-oxide (I) were the top 5 core components of semen strychni.

**Table 1:** Information on 13 active ingredients of strychnine.

Mol ID	Molecule name	OB (%)	DL
MOL003410	Ziziphin_qt	66.95	0.62
MOL000492	(+)-catechin	54.83	0.24
MOL003440	Brucine-N-oxide	52.63	0.38
MOL001476	(S)-Stylopine	51.15	0.85
MOL003433	Brucine-N-oxide	49.17	0.38
MOL003411	Icaride A	48.74	0.43
MOL003432	vomicine	47.56	0.65
MOL000449	Stigmasterol	43.83	0.76
MOL001040	(2R)-5,7-dihydroxy-2-(4-hydroxyphenyl)chroman-4-one	42.36	0.21
MOL003414	Isostrychnine N-oxide (II)	37.33	0.8
MOL003413	Isostrychnine N-oxide (I)	35.45	0.8
MOL003436	Isobrucine	33.58	0.8
MOL003418	Lokundjoside_qt	32.82	0.76

DL = drug-like, OB = oral bioavailability.

### 3.2. Screening of glioma-associated targets

The GeneCards database yielded 10,231 glioma-related targets in total. DisGeNET’s DisGeNET database had 3097 glioma-related targets, and OMIM’s OMIM database contained 195 glioma-related targets. 10,728 glioma targets were found when the targets from the 3 databases were combined and duplicate values were removed.

### 3.3. Construction of PPI network of common target of strychnos and glioma

**Figure 3.**
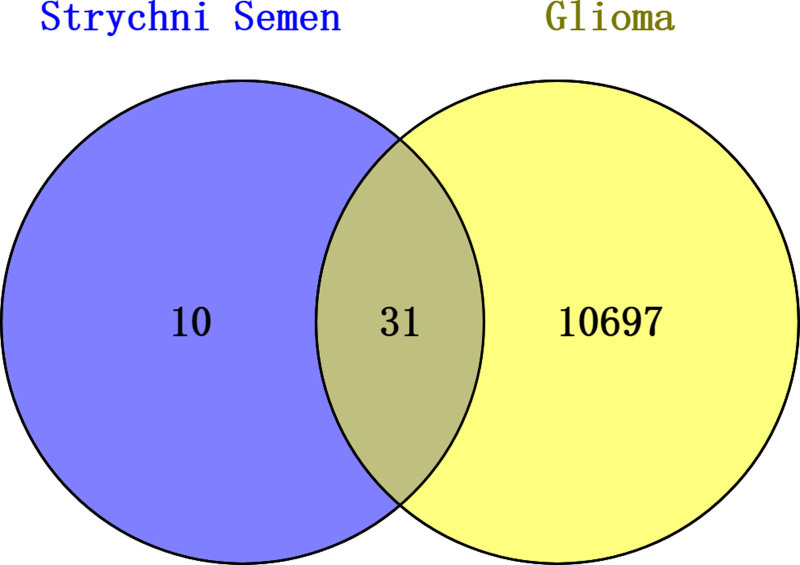
The target of the interaction between strychnine and glioma was obtained by Venny tool.

**Figure 4.**
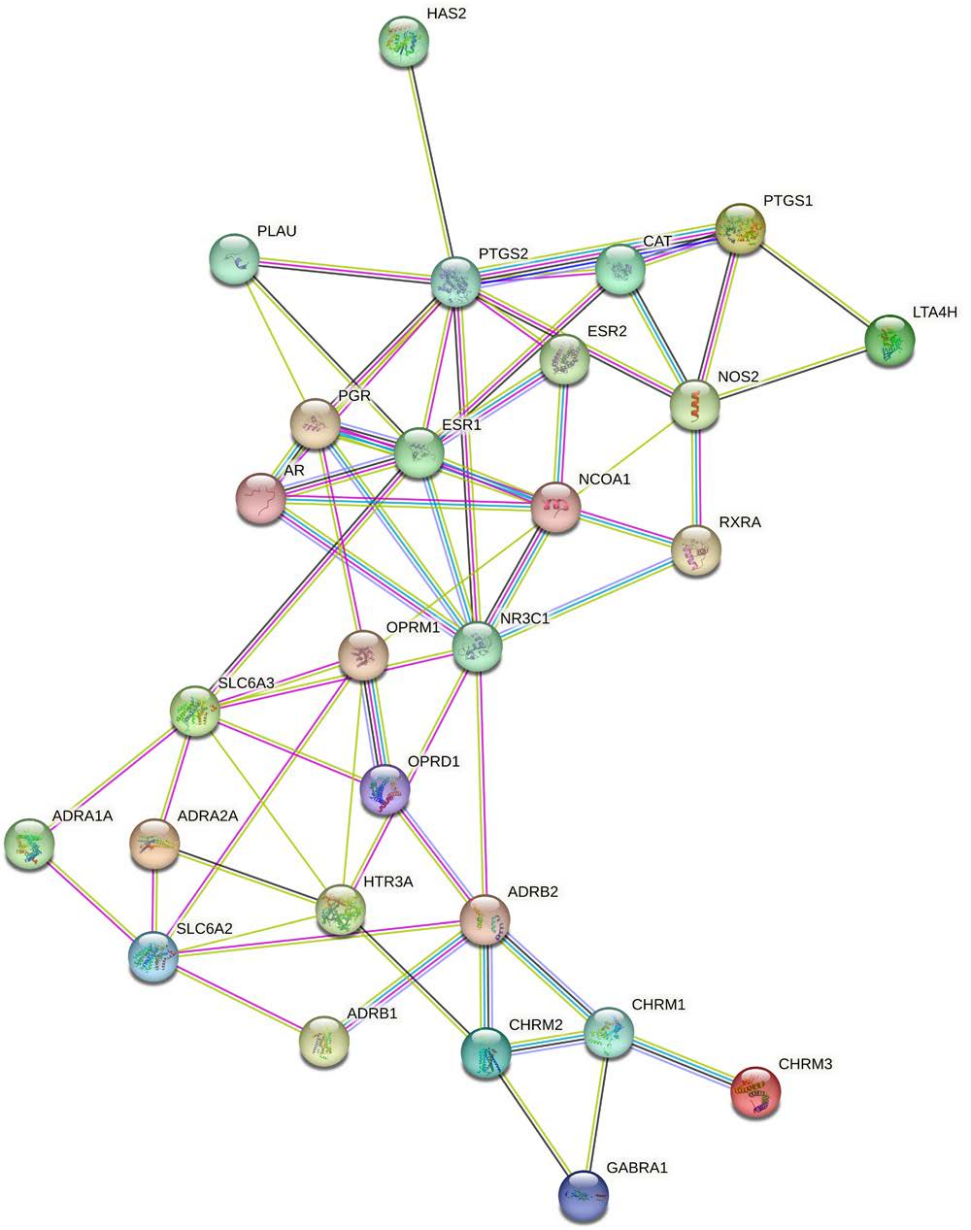
PPI networks. PPI = protein–protein interaction.

Venn diagram analysis revealed 31 interaction sites between semen strychni and gliomas (Fig. 3). To create PPI networks, we loaded 31 interaction targets into the String database and concealed the unconnected nodes (Fig. 4).

### 3.4. GO enrichment and KEGG pathway analysis

The DAVID database was used to import the 31 interaction targets between semen strychni and gliomas for GO enrichment and KEGG pathway analysis. In all, 89 BPs, 44 molecular functions, 32 CC, and 19 KEGG pathways were discovered. *P*-values were used to export and order the data. The 10 elements with the lowest *P*-values for each GO enrichment and KEGG pathway were chosen to create an advanced bubble diagram utilizing bioinformatics. A bigger bubble indicates more genes, whereas a darker bubble indicates a smaller *P*-value.

**Figure 5.**
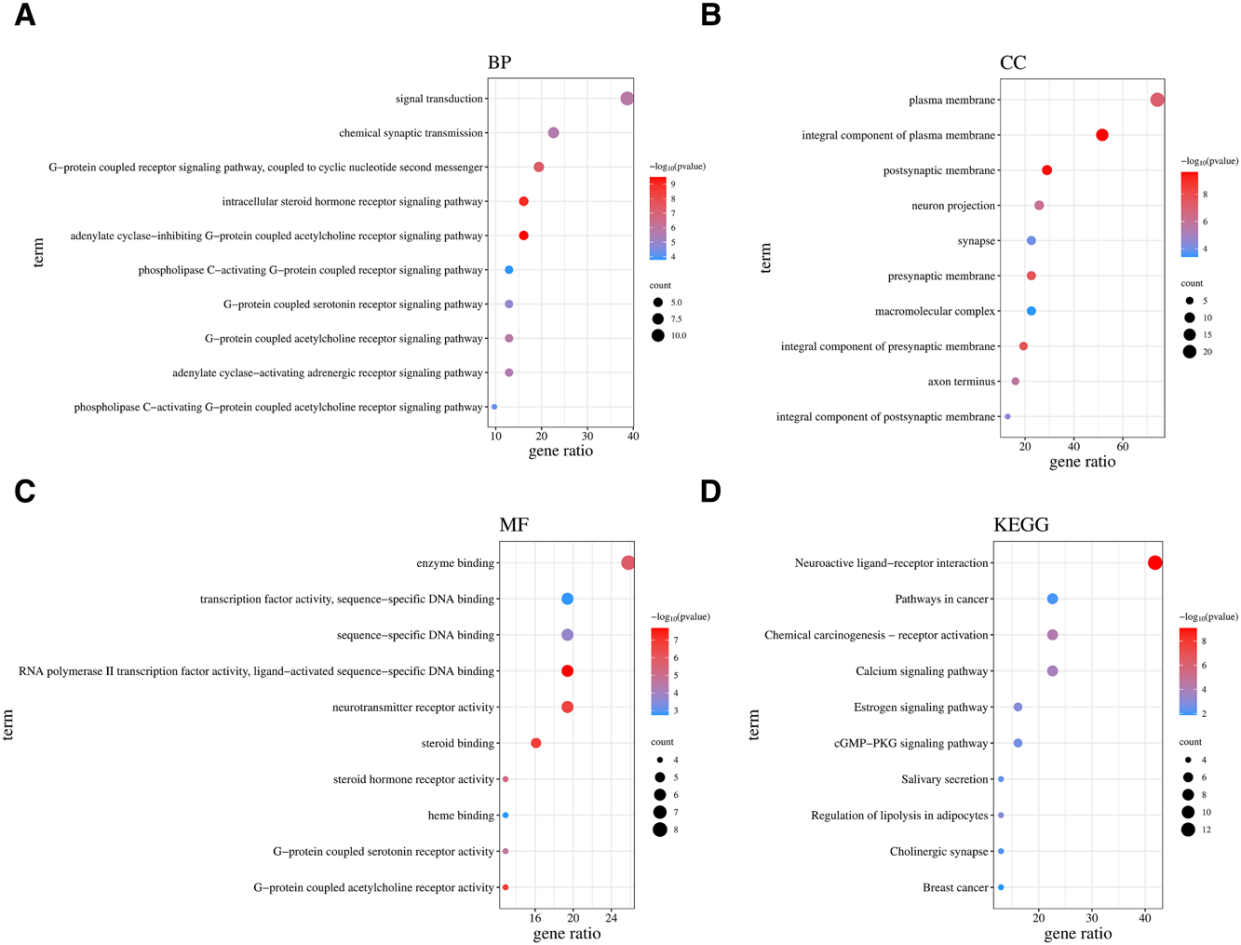
The top 10 enrichment results of GO-BP (A), GO-CC (B), GO-MF (C), and KEGG (D). KEGG = Kyoto Encyclopedia of Genes and Genomes.

The top 10 enrichment results of GO-BP were: adenylate cyclase-inhibiting G-protein coupled acetylcholine receptor signaling pathway, intracellular steroid hormone receptor signaling pathway, G-protein coupled receptor signaling pathway, coupled to cyclic nucleotide second messenger, signal transduction, G-protein coupled acetylcholine receptor signaling pathway, chemical synaptic transmission, adenylate cyclase-activating adrenergic receptor signaling pathway, G-protein coupled serotonin receptor signaling pathway, phospholipase C-activating G-protein coupled acetylcholine receptor signaling pathway, and phospholipase C-activating G-protein coupled receptor signaling pathway (Fig. 5A).

The following were the top 10 enrichment outcomes of GO-CC: The presynaptic membrane, plasma membrane, postsynaptic membrane, integral component of the presynaptic membrane, presynaptic membrane, axon terminus, integral component of the postsynaptic membrane, synapse, and macromolecular complex are all integral membrane components (Fig. 5B).

The top 10 enrichment results of GO-molecular function were RNA polymerase II transcription factor activity, ligand-activated sequence-specific DNA binding, G-protein-coupled acetylcholine receptor activity, steroid binding, neurotransmitter receptor activity, enzyme binding, steroid hormone receptor activity, G-protein-coupled serotonin receptor activity, sequence-specific DNA binding, transcription factor activity, sequence-specific DNA binding, and heme binding (Fig. 5C).

**Figure 6.**
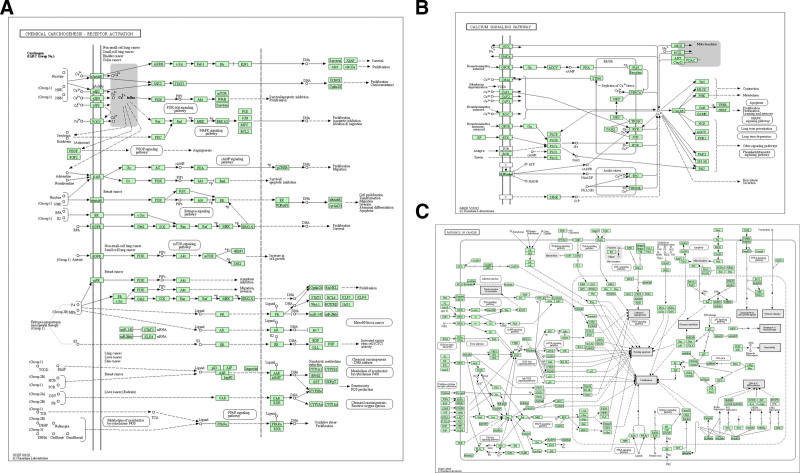
(A) Calcium signaling pathway. (B) Pathways in cancer. (C) The targets-pathways interaction network.

The top 10 KEGG enrichment results were as follows: neuroactive ligand–receptor interaction, chemical carcinogenesis-receptor activation, calcium signaling pathway, regulation of lipolysis in adipocytes, estrogen signaling pathway, cGMP-PKG signaling pathway, salivary secretion, cholinergic synapse, pathways in cancer, and breast cancer (Fig. 5D). The chemical carcinogenesis-receptor activation, calcium signaling pathway, and pathways in cancer are shown in Figure 6.

### 3.5. Construction of a common target-pathway interoperability network

**Figure 7.**
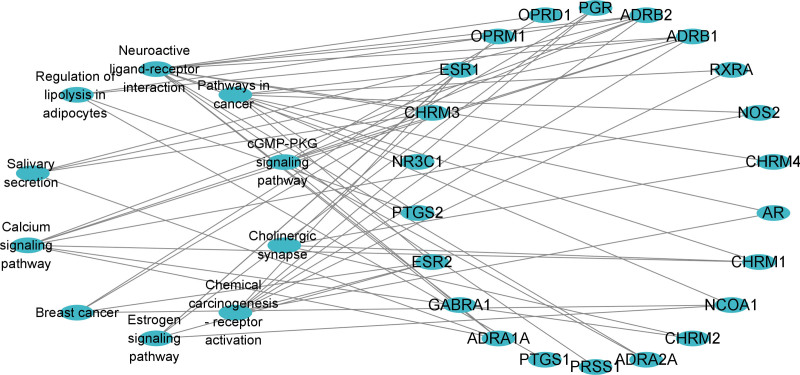
KEGG enrichment analysis of targets in the treatment of glioma using semen strychni. KEGG = Kyoto Encyclopedia of Genes and Genomes.

Cytoscape software was used to build the target-pathway interaction network (Fig. 7). The top 5 main targets of semen strychni for the therapy of glioma were ADRB1, ADRB2, CHRM3, ADRA1A, and ESR1, based on the degree value of the software’s network function analysis.

### 3.6. Molecular docking results

**Figure 8.**
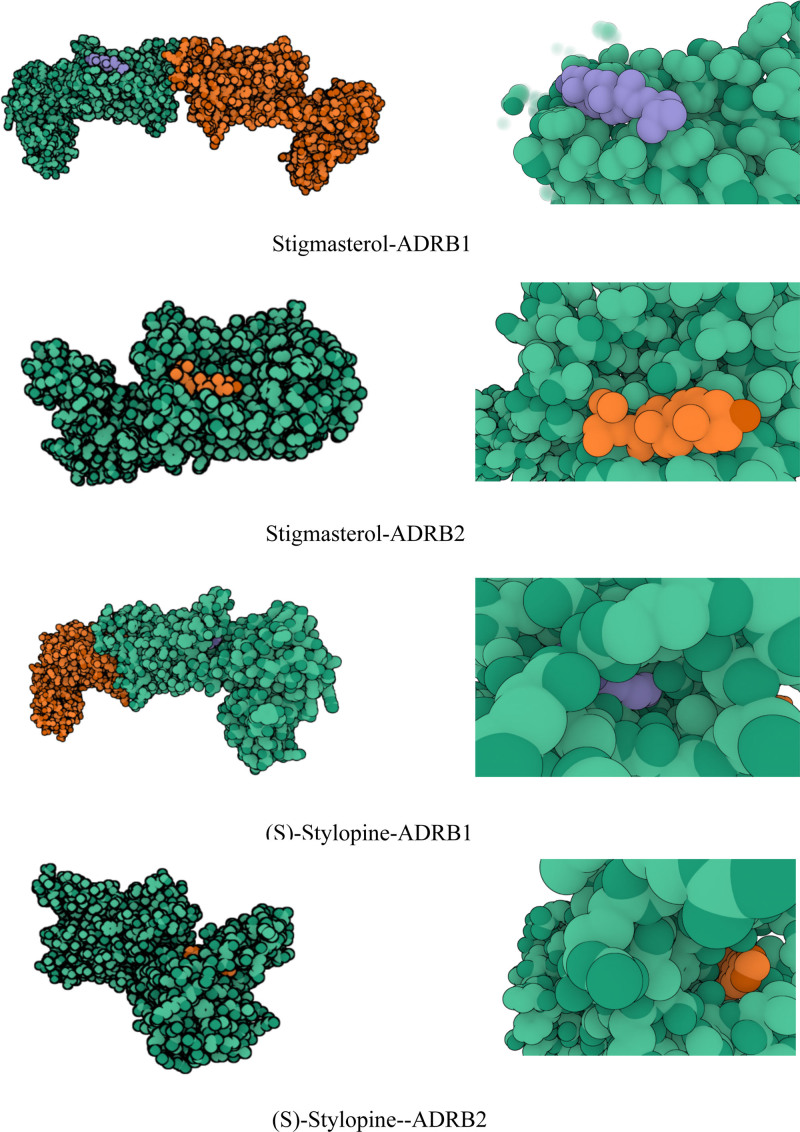
The molecular docking results were visualized by PyMOL.

The top 2 core targets (ADRB1 and ADRB2) and the top 2 core components (stigmasterol and (S)-stylopine) were molecularly docked. The more stable the conformation is, the greater the affinity between the ligand and receptor and the lower the binding free energy between them. The ability of the ligand and receptor to spontaneously interact is demonstrated when the binding free energy is <0. In this work, the molecular docking data for binding energy were displayed (Table 2). The outcomes of the molecular docking are displayed (Fig. 8).

**Table 2:** Molecular docking results.

Compounds	Target	PDB ID	Binding free energy (kcal/mol)
Stigmasterol	ADRB1	7BVQ	−7.616
	ADRB2	6PS2	−8.569
(S)-Stylopine	ADRB1	7BVQ	−9.818
	ADRB2	6PS2	−8.521

ADRB1 = adrenergic receptor, beta 1, ADRB2 = adrenergic receptor, beta 2

### 3.7. Validation of key targets in the TCGA and HPA databases

**Figure 9.**
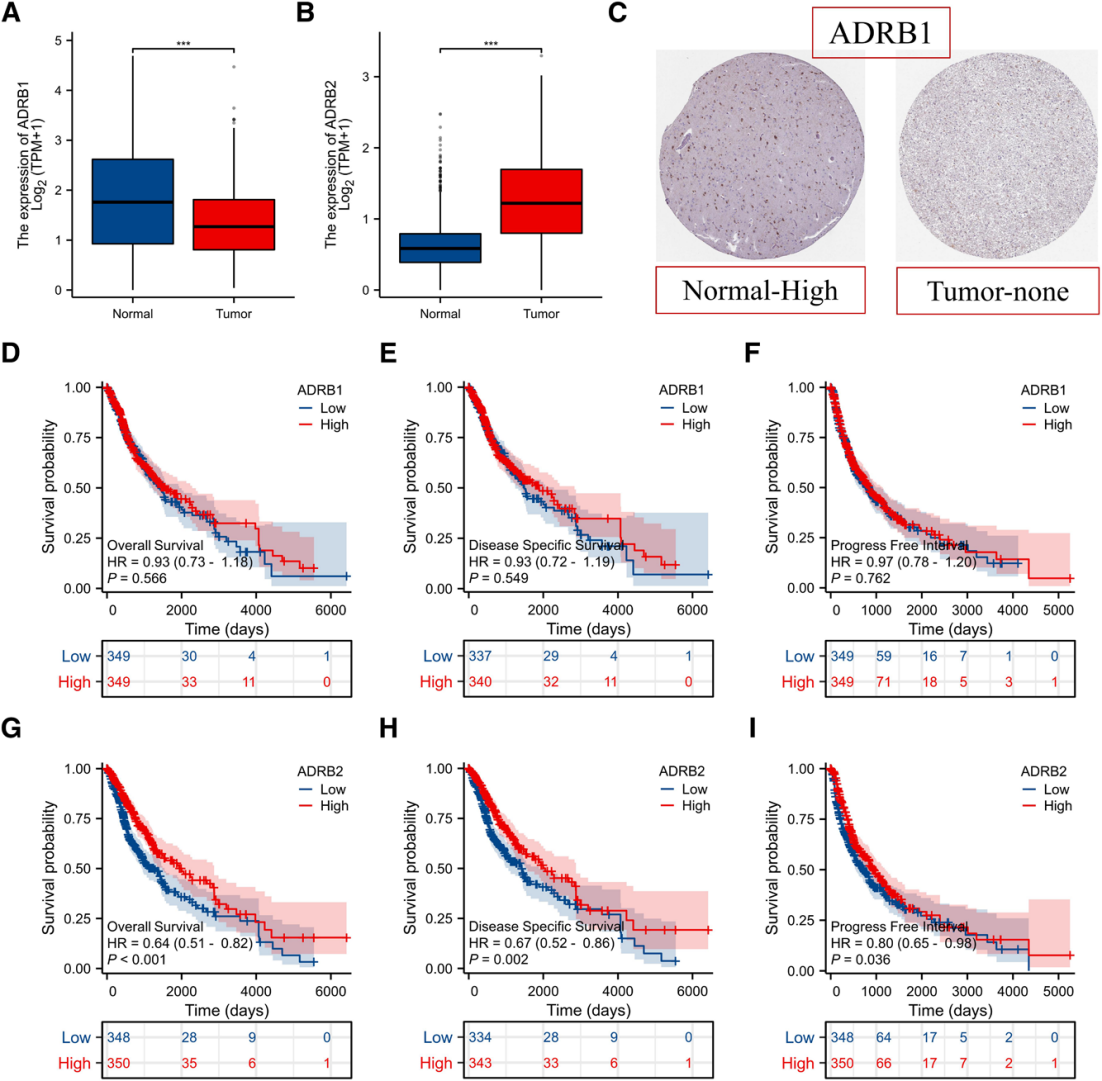
Molecular docking of the active components with key targets.

The expression of key gene mRNA and proteins in tissue samples was studied using the TCGA and HPA databases. The results indicated that the expression of ADBR1 mRNA in glioma tissues was lower than in normal brain tissues (*P* < .05, Fig. 9A), whereas the expression of ADRB2 mRNA was higher (Fig. 9B). From Figures 9C and D, it can be seen that the expression of ADBR1 protein in glioma tissues was lower than in normal brain tissues, and ADBR2 protein was not found in the HPA database. Furthermore, we evaluated the overall survival rate, disease-specific survival, and progression-free interval for key genes in the TCGA database. The results showed that ADBR1 did not have a prognostic significance for the survival of glioma patients (*P* > .05, Fig. 9D–F), while high expression of ADBR2 indicated a good prognosis for glioma patients (*P* < .05, Fig. 9G-I).

### 3.8. Stigmasterol inhibits the malignant progression of U251 cells

**Figure 10.**
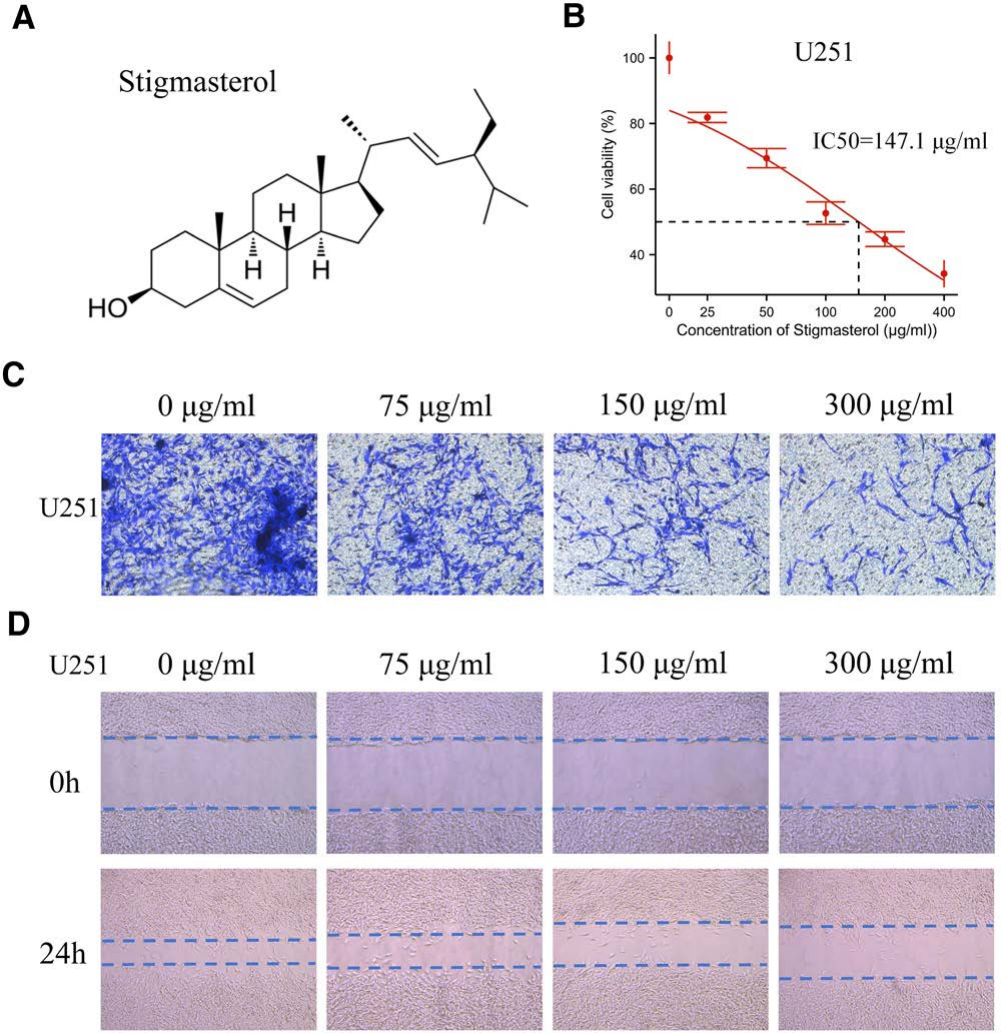
Stigmasterol affects the malignant progression of glioma cells.

To explore the biological function of stigmasterol, we first evaluated the cell viability of U251 cells treated with stigmasterol using the MTS assay. Figure 10A shows the chemical structure of stigmasterol. Moreover, stigmasterol inhibited the viability of U251 cells in a concentration-dependent manner, with an IC50 of 147.1 μg/mL (Fig. 10B). To further investigate whether stigmasterol affects the invasion and migration of glioma cells, we conducted Transwell and wound healing assays. The Transwell assay demonstrated that treatment with a specified concentration of stigmasterol reduced cell invasion through the matrix membrane (Fig. 10C). Furthermore, the wound healing assay indicated that treatment with stigmasterol decreased the migration speed of the cells (Fig. 10D).

## 4. Discussion

Traditional Chinese medicine’s antitumor, analgesic, and anti-inflammatory characteristics make semen strychni a popular treatment.^[18]^ The potential anticancer properties of brucine and vomicin have been demonstrated in several studies.^[19]^ These included stigmasterol, (S)-stylopine, isobrucine, icaride A, and isostrychnine N-oxide (I). In this study, the primary components of semen strychni utilized in the treatment of glioma were preliminary screened utilizing traditional Chinese medicine network pharmacology and molecular docking. A phytosterol called stigmasterol is generated from a variety of herbaceous plants, including tobacco, soybeans, and herbs. Anti-inflammatory, antioxidant, anticancer, and cholesterol-lowering characteristics are all displayed by stigmasterol.^[20]^ Zhao demonstrated how stigmasterol inhibits the Akt/mTOR pathway in gastric cancer cells, which causes apoptosis while also defending against autophagy.^[21]^ In a number of malignancies, stigmasterol has been found to have antitumor effects by encouraging apoptosis, preventing proliferation, metastasis, and invasion, and triggering autophagy in tumor cells. In tumor cells, stigmasterol causes apoptosis by controlling the PI3K/Akt signaling pathway and producing reactive oxygen species in the mitochondria.^[22]^ Stylopine, which Velayutham discovered has the ability to inhibit osteosarcoma cells and control VEGFR2, is a novel therapeutic candidate for the treatment of bone cancer.^[23]^ Analysis of “ADMET”-related factors revealed that (S)-stylopine and isobrucine had high gastrointestinal tract absorption and could pass the blood-brain barrier.^[24]^

The G-protein-coupled receptor family member ADRB1, commonly known as the −1 adrenergic receptor, is a key target for several therapeutic procedures. The co-analysis of tumor mutational load and immune infiltration revealed ADRB1 as a possible biomarker for breast cancer.^[25]^ As a result, activating ADRB1 may protect against neuroinflammatory disorders.^[26]^ In human tumor samples, the expression of ADRB2 was elevated in tumor tissues and linked favorably with tumor size, histological grade, lymph node metastasis, and clinical stage. Chronic stress reversed the tumor growth and metastasis-inhibiting effects of ADRB2 antagonists.^[27]^ Wei et al discovered that ADRB2 controls the tumor immune microenvironment and may be a protective gene in breast cancer.^[28]^ Lehrer et al discovered that in prostate cancer, ADRB1 and ADRB2 were strongly co-expressed with frequently altered genes.^[29]^ By controlling the expression of miR-370-5p/KLF4, inhibiting CHRM3-AS2 expression prevents gliomas from progressing malignantly.^[30]^ According to Chen et al, ADRA1A hypermethylation is a potential biomarker for the detection of hepatocellular carcinoma and may help initiate hepatocellular carcinoma.^[31]^ Patients with hysterocarcinoma have significantly elevated ADRA1A in their peripheral blood, and this expression has been linked to FIGO staging and lymph node metastases. Serum ADRA1A expression may contribute to hysterocarcinoma etiology and metastatic spread, and it may be utilized to gauge survival rates.^[32]^ Methylation of the ESR1 promoter was discovered to be strongly linked with shorter overall and progression-free survival by examining the multiplexed methylation patterns of tumor suppressor genes and clinical outcomes in oligodendroglial malignancies.^[33]^

The KEGG enrichment study led to the major pathways of chemical carcinogenesis, receptor activation, and pathways in cancer being found. These pathways largely affect the formation and prognosis of gliomas by regulating cell proliferation, tumorigenesis, metastasis, angiogenesis, and growth. According to 1 study, PPAR signaling pathways and interactions between neuroactive ligands and receptors are linked to osteosarcoma metastasis.^[34]^ Bioinformatics analysis suggests that pituitary gonadotropic adenoma formation may be facilitated by the activation of the neuroactive ligand-receptor interaction pathway.^[35]^ According to Chen et al’s research, diffuse endogenous pontine gliomas are mostly linked to neuroactive ligand-receptor interactions and a key component of signal transmission, and calcium controls the course of the cell cycle as well as other eukaryotic cell processes.^[36]^ Each cancer characteristic is linked to dysregulation of calcium signaling.^[37]^ In groups IV and II of spinal cord gliomas, the neuroactive ligand-receptor signaling pathway accumulates the majority of differentially expressed genes, demonstrating that it is connected to the evolution of spinal cord gliomas. Similar to this, prior glioma investigations have noted an enrichment of neuroactive ligand–receptor interactions.^[38,39]^ The calcium signaling route has also been linked to the genesis of tumors, and certain cancer cells appear to recruit and modify their calcium ion signaling toolbox for cell growth, invasion, and survival. This remodeling has the potential to be a target for reducing cancer characteristics, although it is not necessarily a source of carcinogenesis. Beyond the cancer cells themselves, the role and significance of calcium ions in tumor growth may also entail the control of the tumor microenvironment.^[40]^ Additionally, it has been discovered that controlling the calcium signaling system can stop cancer cells from growing and encourage their death. This information has been used for the detection and treatment of cancer and has generated novel therapy approaches.^[41]^ The growth of gliomas is tightly associated with the cGMP-PKG signaling pathway. According to studies, this signaling pathway’s activation can encourage fundamental alterations in the growth of tumor cells and lower the survival rate of human glioma cells.^[42]^

Furthermore, the network pharmacology analysis results were validated through in vitro experiments, elucidating the mechanism of action by which stigmasterol treats glioma. MTS assays demonstrated that stigmasterol inhibits the viability of U251 cells in a dose-dependent manner. Tumor metastasis is recognized as a primary characteristic of malignant tumors, emphasizing its significance in the progression and severity of cancer.^[43,44]^ Our study demonstrated that stigmasterol can inhibit the invasion and migration of glioma cells, a finding that aligns with the results of Ma and colleagues’ research.^[45]^

## 5. Conclusions

This study showed that semen strychni cured glioblastoma through several substances, targets, and pathways based on network pharmacological analysis. It also provided a preliminary explanation of the likely mechanism of action of semen strychni in the treatment of gliomas. Stigmasterol, (S)-stylopine, isobrucine, icaride A, and isostrychnine N-oxide (I), which may act on key targets such as ADRB1, ADRB2, CHRM3, ADRA1A, and ESR1, are the main ingredients of semen strychni used to treat gliomas. Chemical carcinogenesis-receptor activation, calcium signaling pathways, and pathways in cancer were the important pathways identified utilizing KEGG enrichment analysis. The outcomes of the molecular docking demonstrated a strong affinity between the central targets and elements. The current work offers encouraging prospects for future research, even though more validations are required to pinpoint the precise mechanism of action of semen strychni.

## Acknowledgments

We would like to extend our heartfelt appreciation to all colleagues and peers who provided invaluable assistance throughout the course of this research endeavor.

## Author contributions

**Data curation:** Xiang Gao.

**Investigation:** Zhixiong Zhang.

**Methodology:** Qiwei Huang.

**Resources:** Wenqu Jiang.

**Software:** Qiwei Huang.

**Validation:** Liang Li, Guofeng Zhu, Yi Zhang.

**Visualization:** Zhixiong Zhang.

**Writing – original draft:** Wenqu Jiang.
